# Label-Free Sensing of Biomolecular Adsorption and Desorption Dynamics by Interfacial Second Harmonic Generation

**DOI:** 10.3390/bios12111048

**Published:** 2022-11-20

**Authors:** Chuansheng Xia, Jianli Sun, Qiong Wang, Jinping Chen, Tianjie Wang, Wenxiong Xu, He Zhang, Yuanyuan Li, Jianhua Chang, Zengliang Shi, Chunxiang Xu, Qiannan Cui

**Affiliations:** 1 State Key Laboratory of Bioelectronics, School of Biological Science and Medical Engineering, Southeast University, Nanjing 210096, China; 2 School of Electronic and Information Engineering, Nanjing University of Information Science and Technology, Nanjing 210044, China

**Keywords:** second harmonic generation, bovine serum albumin, heterointerface, adsorption and desorption, biomolecule

## Abstract

Observing interfacial molecular adsorption and desorption dynamics in a label-free manner is fundamentally important for understanding spatiotemporal transports of matter and energy across interfaces. Here, we report a label-free real-time sensing technique utilizing strong optical second harmonic generation of monolayer 2D semiconductors. BSA molecule adsorption and desorption dynamics on the surface of monolayer MoS_2_ in liquid environments have been all-optically observed through time-resolved second harmonic generation (SHG) measurements. The proposed SHG detection scheme is not only interface specific but also expected to be widely applicable, which, in principle, undertakes a nanometer-scale spatial resolution across interfaces.

## 1. Introduction

Biomolecular activities at interfaces are fundamental phenomena of lives. Interpreting the interfacial dynamics of biomolecules is important for constructing accurate disease models [1,2,3], performing effective drug screening [4,5] and understanding spatiotemporal transport of matter/energy for life systems. As a spatial region with a thickness usually smaller than 10 nm, label-free probing of the interfacial dynamics of biomolecules is challenging. Thus far, only limited label-free probing techniques have been developed, such as surface plasmon resonance [6,7], optical fiber sensors [8], time resolved sum-frequency generation [9,10], surface-enhanced Raman spectroscopy and so on [11,12,13,14,15]. These methods have significantly improved the performance of interfacial biosensing in terms of high sensitivity, high resolution and real-time observation, and have greatly deepened the understanding of the interfacial dynamics at the molecular level.

Surface plasmon resonance microscopy, elegantly utilizing the localized interactions between interfacial electromagnetic fields and biomolecules, possesses molecular-level sensitivity and interfacial spatial resolutions beyond the optical diffraction limit. Inspired by the physical scheme of surface plasmon resonance microscopy, we intend to develop a complementary optical spectrum sensing technique which can realize interfacial biosensing specificity for microfluidic chips in a label-free manner. In our opinion, interfacial second harmonic generation (SHG), a second-order nonlinear optical effect induced by broken inversion symmetry of an interface, is promising to fulfill this goal. Unfortunately, second-order susceptibility of biomolecule interfaces is usually rather small, resulting in a weak SHG signal. In practice, a PMT of a single pixel is usually required to magnify the weak SHG signals. The consequence is that it is difficult to monitor the biomolecular dynamics of an interface in real-time. An alternative way to overcome such difficulty is to significantly increase the power of a fundamental femtosecond laser, which imposes a high risk of damage to the biomaterials and biostructures.

Two dimensional (2D) semiconducting monolayers, such as monolayer MoS_2_, with broken inversion symmetry, possessing large second-order nonlinear optical susceptibility, can produce strong SHG signals under femtosecond laser pulse excitations, which have been comprehensively investigated in the recent decade [16,17,18]. In our opinion, the strong SHG of these 2D semiconducting monolayers can serve as excellent biosensors if biomolecules can interact with them and form heterointerfaces in a liquid environment. Intuitively, the formation of a heterointerface on the surface of 2D semiconducting monolayers can change the inversion symmetry and then lead to a change in SHG signals. Since strong SHG signals of 2D semiconducting monolayers can be readily detected by regular spectrometer or CMOS sensors, it will be possible to develop a real-time sensing technique to probe biomolecule dynamics at 2D interfaces.

To establish progress, we report a label-free interfacial biomolecular sensing technique by monitoring biomolecular adsorption and desorption processes on the surface of monolayer MoS_2_ through time-resolved SHG. Chitosan nanoclusters and bovine serum albumin (BSA) molecules have been used to form effective Coulomb attractions with the negatively charged surface of monolayer MoS_2_. By measuring the SHG intensity changes as a function of time, we realize the label-free real-time sensing of biomolecular adsorption and desorption processes all-optically in liquid environments. Our results open new avenues of label-free interfacial biosensing, taking advantage of the strong optical SHG of monolayer 2D semiconductors.

## 2. Results and Discussion

Monolayer MoS_2_ is an ideal platform to construct biosensors for interfacial molecule adsorption and desorption processes, considering that the monolayer MoS_2_ lattice undertakes a sub-nanometer thickness with broken inversion symmetry. Inspired by the pioneering work of strong SHG observations in monolayer MoS_2_ in 2013 [16], monolayer MoS_2_ can be considered as a sub-nanometer thick nonlinear optical source emitting SHG with extremely space-confined dipole moments, which can facilitate the interfacial sensing and imaging of ultrahigh spatial resolution across interfaces. Moreover, large specific surface areas and the abundant binding sites of monolayer MoS_2_ can further enable effective interaction with biomolecules. To be specific, negatively charged surfaces of monolayer MoS_2_ samples grown by chemical vapor deposition (CVD) [19,20,21,22,23] are expected to induce strong Coulomb attractions in liquid environments for positively charged biomolecules, which, in our opinion, are very promising for realizing label-free, real-time sensing of molecule adsorption and desorption processes.

**Figure 1:**
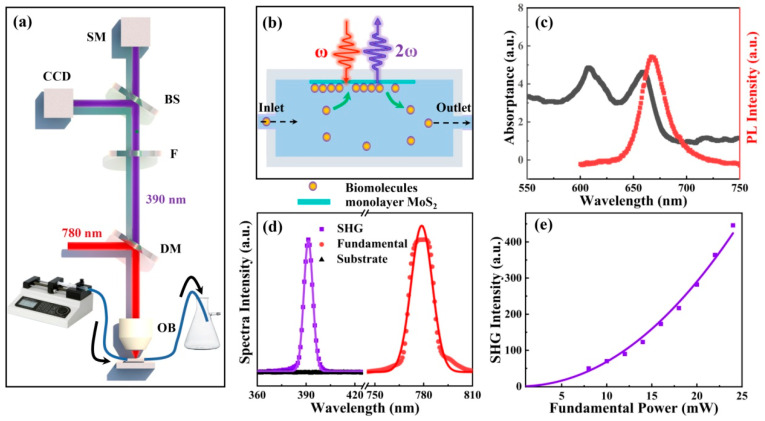
(**a**) Experimental setup of SHG detection with a microfluidic chip. SM: Spectrometer, BS: Beam splitter, F: Filter, DM: Dichroic mirror, OB: Objective lens. (**b**) Schematic diagram of the microfluidic chip. (**c**) Optical absorbance and photoluminescence (PL) spectra of monolayer MoS_2_. (**d**) The fundamental laser (red) and SHG spectra of monolayer MoS_2_ (purple) and sapphire substrate (black). (**e**) Fundamental power dependence of SHG in monolayer MoS_2_.

Figure 1a shows the experimental setup detecting the SHG spectra of monolayer MoS_2_ embedded in a microfluidic chip. The wavelength of a fundamental laser is centered at 780 nm, with a pulse width of about 60 fs and a repetition frequency of about 80 MHz. The fundamental laser is reflected by the dichroic mirror and then focused on the MoS_2_ monolayer by the objective lens (NA = 0.55). SHG of the monolayer MoS_2_ is collected by the same objective lens. A short-pass filter is deployed behind a dichroic mirror to eliminate the reflected fundamental residual. The SHG signals are simultaneously sent to a spectrometer and a CCD by a beam splitter. In our measurements, the power of the fundamental laser was low enough to avoid optical damage of monolayer MoS_2_. The fine structure of the microfluidic chip is illustrated in Figure 1b. The main body of the microfluidic chip is fabricated by 3D printing with an optical glass window on top. A sapphire substrate containing monolayer MoS_2_ is attached to the optical glass window. The microfluidic channel enables unidirectional flow of liquid solutions of biomolecules to form a laminar flow. As a result, a homogeneous 3D fluid–2D solid interaction is constructed to facilitate adsorption and desorption of biomolecules on the surface of monolayer MoS_2_. Monolayer MoS_2_ grown by CVD on double-sided polished sapphire substrates (Six Carbon Technology, Shenzen, China) were used as received. To confirm the monolayer nature of these samples, optical characterizations were carried out before microfluidic experiments. Figure 1c presents optical absorption and photoluminescence (PL) spectra of monolayer MoS_2_. Lorentzian fitting of the PL spectrum points to a resonant peak centered at about 669 nm, which agrees well with the A-exciton resonance peak of the optical absorption spectrum [24,25]. Employing the experimental setup of Figure 1a, we measured SHG (centered at 390 nm) and fundamental (centered at 780 nm) spectra of monolayer MoS_2_, as indicated by the purple squares and red dots in Figure 1d, respectively. Solid lines are Gaussian fittings. Full width at half maxima (FWHM) of fundamental and SHG were fitted to be about 12.8 nm and 5.5 nm. Meanwhile, no SHG signals were observed when the fundamental was focused on the substrate, as indicated by the spectrum of sapphire (black triangles) in Figure 1d. Fundamental power dependence of SHG spectra was obtained by varying the power of the 780-nm femtosecond laser, and the result is presented in Figure 1e. Fitting with a square function (solid purple line) matches well with experimental SHG results (purple dots), suggesting a quadratic dependence of SHG power with respect to fundamental power [16]. These observations validate that our MoS_2_ samples are monolayers, and strong SHG can be readily recorded by our experimental setup equipped with a regular spectrometer.

**Figure 2:**
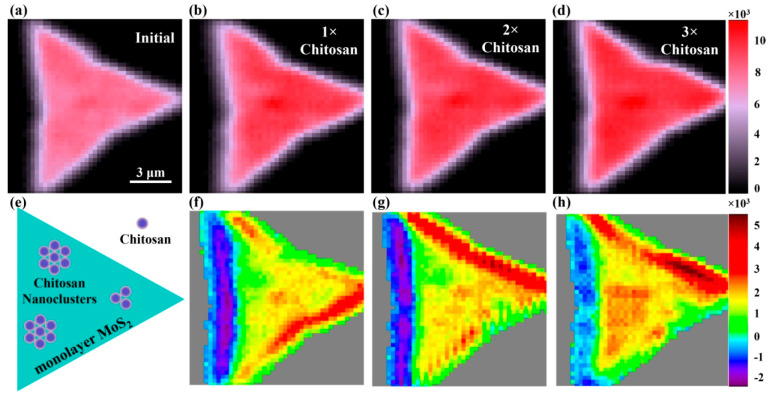
SHG mapping of monolayer MoS_2_ adsorbing chitosan nanoclusters. (**a**) SHG mapping of monolayer MoS_2_ (**a**) without chitosan nanoclusters and (**b**–**d**) with adsorbed chitosan nanoclusters of increasing concentrations. (**e**) Schematic diagram of chitosan nanocluster adsorption on surface of monolayer MoS_2_. (**f**–**h**) Differential SHG images of (**b**–**d**) with respect to (**a**).

To justify the electrostatic adsorption effect of monolayer MoS_2_, we employed positively charged chitosan to interact with monolayer MoS_2_. Chitosan was dissolved into a water solution of acetic acid, configuring a solution with a mass fraction of 0.8 mg/mL. Before adding the chitosan solution, a pixel-to-pixel SHG mapping of a monolayer MoS_2_ sample in the air was taken by scanning a 2D translation stage (Physik Instrumente, P-51, Karlsruhe, Germany). The SHG image of this sample was presented in Figure 2a. The spatial scanning step was set at 300 nm, and the grey value of each pixel was obtained by integrating the SHG spectrum counts within a wavelength ranging from 370 nm to 410 nm. For monolayer MoS_2_ region, strong SHG leads to a distribution of triangle reflecting the spatial profile of monolayer MoS_2_ lattice. For the substrate region, the absence of SHG points to a black background. Subsequently, 1µL of chitosan solution (one drop) was added onto the same monolayer MoS_2_ sample. When the solution completely evaporated at room temperature, pixel-to-pixel SHG mapping of the monolayer MoS_2_ sample was repeated. The obtained SHG image was presented in Figure 2b. Similarly, by repeating the procedures of dropping, drying and SHG mapping, Figure 2c,d are SHG images of the same monolayer MoS_2_ sample covered by two and three drops of chitosan solutions, respectively. We anticipate that the electrostatic adsorption process can be initiated by the Coulomb forces between the negatively charged monolayer and chitosan. Accumulated amounts of chitosan are expected to form chitosan nanoclusters on the surface of monolayer MoS_2_, as illustrated by the schematic diagram in Figure 2e. By carefully comparing the SHG images before (Figure 2a) and after (Figure 2b–d) adding chitosan solutions, it is clear that adsorbed chitosan nanoclusters on monolayer MoS_2_ can enhance SHG’s intensity. To directly reveal these differences, we subtracted the SHG image in Figure 2a from that in Figure 2b–d. The corresponding differential SHG images are plotted in Figure 2f–h. These differential SHG images present randomly distributed SHG enhancements, suggesting adsorbed chitosan nanoclusters were randomly distributed, as well. We expect that this phenomenon originated from the process wherein a solution dropping action causes a rearrangement of molecules on the surface of monolayer MoS_2_. Rather, the differential SHG intensity shows an increasing trend from Figure 2f to Figure 2h, as added amounts of chitosan were increased. Especially, for certain edge regions of monolayer MoS_2_, SHG enhancement effects turn out to be stronger, suggesting that the edge region with more charged active sites tends to facilitate chitosan adsorptions [26].

**Figure 3:**
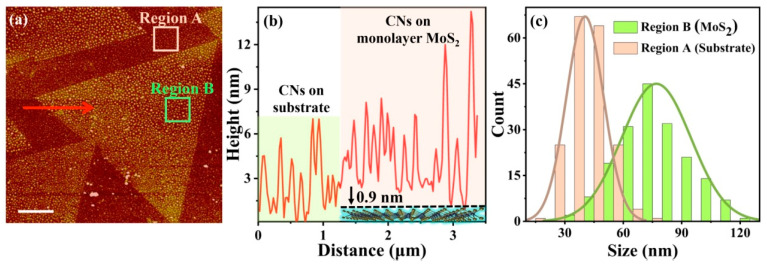
(**a**) Atomic force microscopy (AFM) image of monolayer MoS_2_ with adsorbed chitosan nanoclusters (CNs). The scale bar is 2 μm. (**b**) Height profile along the red arrow in (**a**). (**c**) Size histogram of chitosan nanoclusters distributed on the surface of monolayer MoS_2_ and sapphire substrate.

To visualize the adsorbed chitosan nanoclusters on the surface of monolayer MoS_2_, we measured the AFM image after dropping and drying a chitosan solution for monolayer MoS_2_ samples on the sapphire substrate. As shown in Figure 3a, it is clear that chitosan nanoclusters adsorbed on monolayer MoS_2_ (triangle region) form many white dots. Furthermore, on the sapphire substrate, some chitosan nanoclusters can still be absorbed, but their density of distribution as well as size are smaller than the case of monolayer MoS_2_. The physical reason is that positively charged chitosan molecules tend to be more efficiently adsorbed by the negatively charged monolayer MoS_2_ through attractive Coulomb forces [19,20,21,22,23]. The height profile along the red arrow in Figure 3a is plotted in Figure 3b. Observed height values of CNs on the sapphire substrate (distance range: from 0 to 1.4 µm) suggest an averaged thickness of about 7 nm. It is obvious that height values of CNs on the monolayer MoS_2_ (distance range: from 1.4 to 3.5 µm) could be as large as about 13 nm. The measured thickness of monolayer MoS_2_ is less than 1 nm (about 0.9 nm) according to Figure 3b, which agrees well with previous measurement [27]. In addition, size distributions of adsorbed chitosan nanoclusters on monolayer MoS_2_ and on the sapphire substrate were analyzed, as shown in Figure 3c, where two representative regions, marked in Figure 3a, were selected. Histograms of adsorbed chitosan nanoclusters in red and green of Figure 3c are statistics of region A and B of Figure 3a, respectively. After fitting these histograms with a Gaussian function, it turns out that the averaged diameter of adsorbed chitosan nanoclusters on substrate is about 41 nm. In comparison, on monolayer MoS_2_, the averaged diameter of adsorbed chitosan nanoclusters is about 78 nm, validating that Coulomb attraction forces between monolayer MoS_2_ and chitosan nanoclusters enhance the adsorption processes. The size FWHM of adsorbed chitosan nanoclusters on monolayer MoS_2_ is about 42 nm, which is about twice that (about 22 nm) on the substrate. At this point, we can conclude that chitosan nanoclusters can be effectively adsorbed on monolayer MoS_2_ by electrostatic attractions and mediate SHG intensity after drying. However, whether such an interfacial adsorption effect can induce enough SHG intensity change for biomolecules flowing in a liquid environment is still unknown.

**Figure 4:**
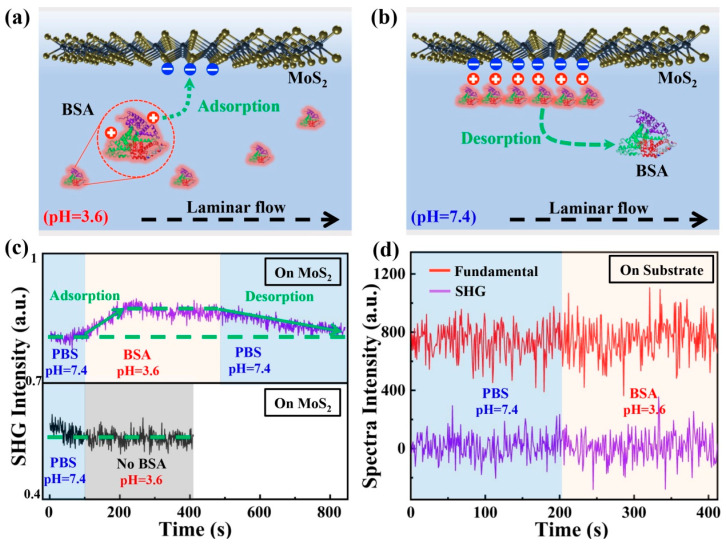
(**a**,**b**) Schematic diagram of BSA adsorption and desorption processes on surface of monolayer MoS_2_. (**c**) Time-resolved SHG signals (purple) of BSA adsorption and desorption processes on monolayer MoS_2_. The lower panel is time-resolved SHG signals (black) when there is no BSA. (**d**) Time-resolved fundamental (red) and SHG (purple) signals of substrate.

To demonstrate the feasibility of our SHG technique towards real-time sensing for flowing biomolecules in liquid environments, we selected bovine serum albumin (BSA) molecules and performed time-resolved SHG spectra measurements employing the experimental setup in Figure 1a,b. The proposed experimental schemes of BSA adsorption and desorption are presented in Figure 4a,b, respectively. Simply speaking, protonated BSA molecules are positively charged, so that the adsorption process is expected when a negatively charged monolayer MoS_2_ tends to apply attractive Coulomb forces. Then, by controlling the pH of the liquid environment, protonated BSA molecules can gain electrons, and positive charges of adsorbed BSA molecules will be neutralized. Laminar flow in the microfluidic channel will take away these interfacial BSA molecules and trigger a desorption process. Before placing the monolayer MoS_2_, the microfluidic chip and tubes were carefully cleaned. The BSA solution (1 μg/mL) was configured in PBS buffer solution (pH = 3.6) using BSA (5%, Yuanye Bio-Technology, Shanghai, China). The power of the fundamental laser was fixed at 8 mW. By focusing the fundamental laser tightly on the center of a monolayer MoS_2_ sample by a 50× objective lens (NA = 0.55), we ensured that the size of the focal spot (1.7 µm) was much smaller than the lateral size of the monolayer MoS_2_ sample (about 15 µm). By finely tuning the axial position of monolayer MoS_2_ sample, we maximized the intensity of SHG spectrum recorded by the spectrometer. A computer program was coded to record the SHG spectra every 500 ms, which integrated all non-zero SHG counts between 370 nm and 410 nm.

In our measurements, a fluid pump sent solutions into the microfluidic channel at a constant flowing rate of 22 μL/s. In the beginning, we flushed the microfluidic chip with a PBS solution (pH = 7.4) until the SHG signal of monolayer MoS_2_ became stable in flowing conditions, as indicated by the time-resolved SHG signals (purple spectra) before 90 s in Figure 4c. At the 90 s mark, the BSA solution (pH = 3.6) was sent into the microfluidic channel. The intensity of SHG signals started to increase and, approximately, maintained a constant after the 200 s point. The increasing evolution of SHG signals between 90 s and 200 s is caused by the BSA molecule’s adsorption on the surface of monolayer MoS_2_. Then, at 480 s, the PBS solution (pH = 7.4) was sent to trigger a BSA molecule desorption process. Interestingly, the intensity of SHG signals started to decrease and, eventually, recovered to a constant magnitude at about 850 s, which is equal to the scenario when no BSA was added (before the 90 s mark). Furthermore, a control experiment was performed by shifting the fundamental laser focus onto a nearby monolayer MoS_2_ sample and replacing the BSA solution with a PBS solution without BSA molecules, while other experimental conditions were kept the same. As indicated by the lower panel of Figure 4c, at 90s, the intensity of SHG signals (black spectra) when the fundamental laser was focusing on monolayer MoS_2_ remained a constant. This result strongly validates that ions or other molecule components in the PBS solutions would not induce a detectable intensity change of monolayer MoS_2_ SHG signals for our experimental setup. The baseline decrease is attributed to the lattice orientation difference in CVD MoS_2_ from sample to sample. To evaluate the contributions of adsorbed BSA molecules to the refractive index change as well as intensity change of SHG signals, we focused the fundamental laser on the sapphire substrate. By replacing the short-pass optical filters in front of the spectrometer, a small portion of the fundamental laser (780 nm) was allowed to pass. Hence, we can measure fundamental and SHG signals at the same time. The BSA adsorption experiments were repeated by adding BSA solutions at 200 s. As shown in Figure 4d, the spectra intensity of fundamental (780 nm) remained a constant after adding BSA molecules, ruling out the possible effect of interfacial refractive index change. More importantly, the spectra intensity of SHG remained zero, indicating that SHG contributions of the sapphire substrate as well as adsorbed BSA generated can be neglected compared with the monolayer MoS_2_. The low SHG conversion efficiency of sub-nanometer thick monolayer MoS_2_ and high noise-level of our spectrometer lead to a relatively low signal to noise ratio. This issue can be further improved by increasing the integration time of each SHG spectrum and optimizing the design of microfluidic chips.

To address the physical mechanism of observed SHG signal changes accompanying the BSA adsorption process, we can consider the second-order polarization of the interface with the follow model [28,29]: $E_{2\omega }\propto P^{\left(2\right)}=\chi^{\left(2\right)}\;E_{\omega }E_{\omega }+\chi^{\left(3\right)}\;E_{\omega }E_{\omega }\emptyset_{0}$, where $\emptyset_{0}$ is the interfacial electric field and *χ*^(2)^ and *χ*^(3)^ are the second-order optical susceptibility of monolayer MoS_2_ and third-order optical susceptibility of the interface, respectively. In our case, we believe that the spatial distribution of adsorbed BSA molecules on the surface monolayer MoS_2_ is random. Specifically, since the initial charge distribution of monolayer MoS_2_ is inhomogeneous in a 2D plane defined by the flat substrate, the orientations and 3D stacking orders of adsorbed BSA molecules are expected to be highly random. As a result, directions of interfacial electric fields between the monolayer MoS_2_ and adsorbed BSA molecules in disorder will no longer be strictly perpendicular to the 2D plane. Therefore, the angle between the wave vector of the fundamental laser and the direction of interfacial electric fields will not be zero, so that the $\emptyset_{0}$ term can induce a non-zero second-order polarization field. When BSA molecules are dynamically adsorbed, the total magnitude of second-order polarization fields formed by superposition of polarization fields from monolayer MoS_2_ and interfacial electric fields will depend on their initial phase difference.

## 3. Conclusions

We have comprehensively demonstrated that the interfacial SHG of monolayer MoS_2_ can be utilized for label-free biomolecule sensing. Through static SHG mapping experiments, we show that the intensity of SHG in monolayer MoS_2_/adsorbed chitosan nanocluster heterostructures can be mediated due to electrostatic attractions. With a time-resolved SHG measuring system equipped with a microfluidic chip, we further realize label-free sensing of BSA adsorption and desorption dynamics in real time through the SHG intensity change of monolayer MoS_2_ in liquid environments, which has been tailored by Coulomb interactions between BSA molecules and monolayer MoS_2_. Our work provides a complementary mean of label-free interfacial biomolecule sensing, which, in principle, undertakes molecular-level spatial resolution across the interfaces for applications including, but not limited to, in vitro medicine evaluation.

## Data Availability

Not applicable.
